# High frequency of pathogenic germline variants within homologous recombination repair in patients with advanced cancer

**DOI:** 10.1038/s41525-019-0087-6

**Published:** 2019-06-21

**Authors:** Birgitte Bertelsen, Ida Viller Tuxen, Christina Westmose Yde, Migle Gabrielaite, Mathias Husted Torp, Savvas Kinalis, Olga Oestrup, Kristoffer Rohrberg, Iben Spangaard, Eric Santoni-Rugiu, Karin Wadt, Morten Mau-Sorensen, Ulrik Lassen, Finn Cilius Nielsen

**Affiliations:** 1 Center for Genomic Medicine, Rigshospitalet, Copenhagen, Denmark; 2 The Phase I Unit, Department of Oncology, Rigshospitalet, Copenhagen, Denmark; 3 Department of Pathology, Rigshospitalet, Copenhagen, Denmark; 4 Department of Clinical Genetics, Rigshospitalet, Copenhagen, Denmark

**Keywords:** Cancer genetics, Cancer, Genetic testing

## Abstract

Genomic screening of cancer patients for predisposing variants is traditionally based on age at onset, family history and type of cancer. Whereas the clinical guidelines have proven efficient in identifying families exhibiting classical attributes of hereditary cancer, the frequency of patients with alternative presentations is unclear. We identified and characterized germline variants in 636 patients with advanced solid cancer using whole exome sequencing. Pathogenic and likely pathogenic germline variants among 168 genes associated with hereditary cancer were considered. These variants were identified in 17.8% of the patients and within a wide range of cancer types. In particular, patients with mesothelioma, ovarian cancer, cervical cancer, urothelial cancer, and cancer of unknown primary origin displayed high frequencies of pathogenic variants. Variants were predominantly found in DNA-repair pathways and about half were within genes involved in homologous recombination repair. Twenty-two *BRCA1* and *BRCA2* germline variants were identified in 12 different cancer types, of which 10 (45%) were not previously identified in these patients based on the current clinical guidelines. Loss of heterozygosity and somatic second hits were identified in several of the affected genes, supporting possible causality for cancer development. A potential treatment target based on the pathogenic germline variant could be suggested in 25 patients (4%). The study demonstrates a high frequency of pathogenic germline variants in the homologous recombination pathway in patients with advanced solid cancers. We infer that genetic screening in this group of patients may reveal high-risk families and identify patients with potential PARP inhibitor sensitive tumors.

## Introduction

Precision medicine using next-generation sequencing (NGS) is rapidly being implemented in clinical oncology aiming to identify actionable tumor aberrations to guide trial allocation.^1–5^ Different strategies based on tumor profiling are employed to select patients for clinical trials but so far the significance of germline findings is incompletely understood. The American College of Medical Genetics and Genomics/Association for Molecular Pathology (ACMG/AMP) are recommending return of germline findings in 59 genes in clinical exome and genome sequencing, of which 29 are associated to hereditary cancer.^6,7^ However, there are still a number of unresolved issues regarding handling of returns and clinical implementation.

Depending on the patient cohort and the method used for identification of germline variants previous studies have reported a prevalence of putative pathogenic germline variants in 4.3–17.5% of cancer patients.^8–11^ In particular, pathogenic variants in the two high penetrance genes *BRCA1* and *BRCA2*, known to predispose to breast and ovarian cancer, are frequently observed.^12^ Thus, 72% of women carrying a *BRCA1* mutation and 69% with a *BRCA2* mutation will develop breast cancer by the age of 80, while up to 44% with a *BRCA1* mutation and 17% with a *BRCA2* mutation will develop ovarian cancer.^13^ Mutations in *BRCA1* and *BRCA2* however also increase the risk of other cancers, including pancreatic,^14^ fallopian tube, and peritoneal cancer.^15,16^ Men with *BRCA2* mutations, and to a lesser extent *BRCA1* mutations, are also at increased risk of breast^17^ and prostate cancer.^18,19^ Several other genes have been linked to hereditary cancer. The mismatch repair (MMR) genes *MLH1*, *MSH2*, *MSH6*, and *PMS2* are associated with hereditary nonpolyposis colorectal cancer (HNPCC)^20^ and *APC* mutations are found in individuals with familial adenomatous polyposis (FAP).^21^ Mutations in *TP53*, *PTEN*, *CDH1*, *CHEK2*, *ATM*, *RAD51D*, *PALB2*, and *FANCM* have been associated with increased risk of breast cancer^22,23^ or prostate cancer,^19^ although with varying penetrance. In particular loss-of-function germline variants in *PALB2* increase the risk of breast cancer before 40 years of age eight times^24^ and heterozygous mutations within the *FANCM* gene were recently demonstrated to be associated with familial breast cancer, in particular for early-onset or triple-negative breast cancer.^23^ Furthermore, bi-allelic inherited germline variants in ataxia telangiectasia mutated (*ATM*) result in the multisystem disorder ataxia telangiectasia syndrome, which manifests with progressive neurological disease and an increased predisposition to lymphoid, gastric, breast, central nervous system, and skin cancer, as well as other cancers.^25,26^ Studies have shown a moderate increased risk of breast cancer in heterozygote carriers of pathogenic *ATM* variants.^27^ Studies on germline ataxia-telangiectasia and Rad3-related (*ATR*) mutations with respect to cancer susceptibility have mainly been inconsistent or inconclusive.^28^ Thus, the role of *ATR* variants for cancer susceptibility remains unclear.

Most of the known cancer susceptibility genes are involved in maintenance of genomic integrity, safeguarding DNA from mutations that ultimately could lead to malignancy. BRCA1 and BRCA2 proteins are key players in the molecular events following double-stranded DNA damage and homologous recombination (HR),^29^ while ATM, ATR, CHK1, CHK2, and Tumor suppressor p53 proteins are all central players in sensing and orchestrating the checkpoint signaling from double strand breaks (DSBs) to DNA repair.^30^ In contrary, the MMR system recognizes and repairs DNA errors from mismatched nucleotides with MSH proteins being key players in recognizing and initiating the MMR repair process.^31^

The response to various therapies are affected by the hereditary predispositions. In case of *BRCA1* or *BRCA2*, mutations improve responsiveness to platinum-based chemotherapy, as well as poly(ADP-ribose) polymerase inhibitor (PARPi) treatment.^32^ In particular, PARPi treatment of patients with ovarian and breast cancer carrying germline *BRCA1/2* mutations has shown an exceptional increase in response rate and progression-free survival, illustrating the first clinical proof for the concept of synthetic lethality.^33^ These results have led to clinical approval of PARPi in ovarian cancer, and in January 2018, also for the treatment of germline *BRCA1/2* mutated metastatic breast cancers, thereby becoming the first targeted therapy for patients with breast cancer carrying *BRCA1/2* mutations.^34^ In addition, prostate cancers with somatic or germline variants in DNA-repair genes including *BRCA2* and *ATM* displayed a high response rate to PARPi treatment.^35^ Finally, mutations in MMR genes are associated with microsatellite instability and high mutational burden which confer a better response to anti-PD-1 immunotherapy.^36,37^

In this study we examined the clinical significance of germline screening in patients with advanced cancer. We investigated a cohort of 636 advanced cancer patients for pathogenic germline variants to gain insights into possible new associations between germline variants, cancer types and molecular pathways.

## Results

### Germline variants: frequency and characteristics

A total of 636 patients with advanced cancer referred to the Phase 1 unit were included in the study. The patient characteristics are summarized in Table 1. Out of these patients, 17.8% (*n* = 113) were shown to have a pathogenic or likely pathogenic (hereafter collectively referred to as pathogenic) germline variant in at least one of the 168 cancer-associated genes tested (Supplementary Table 1; excluding heterozygous variants in genes known to have a recessive inheritance pattern) (Table 1). Four patients carried two pathogenic variants while two patients carried three pathogenic variants (Supplementary Table 2). In addition, 16 patients were heterozygous for pathogenic variants in genes known to have a recessive pattern of inheritance, i.e., the colorectal cancer (CRC) predisposition genes *MUTYH* and *NTHL1* (Supplementary Table 3). As we did not identify any patients with homozygous variants nor potentially compound heterozygous variants, all presumed genes associated with recessive conditions were excluded from further analysis.

**Table 1:** Patient characteristics

	Total (*n* = 636)	Patients with pathogenic variant (*n* = 113)
*Gender*		
Female	321 (50%)	58 (51%)
Male	315 (50%)	53 (49%)
*Age at diagnosis*		
Median, range	57 (16–82)	54 (26–77)
History of prior cancer^a^	103 (16%)	24 (22%)
*Tumor type*		
Colorectal cancer	141 (22%)	27 (24%)
Breast cancer	85 (13%)	16 (14%)
Bile duct cancer	47 (7%)	8 (7%)
Pancreatic cancer	43 (7%)	7 (6%)
NSCLC	33 (5%)	4 (4%)
Prostate cancer	26 (4%)	3 (3%)
Ovarian cancer	23 (4%)	7 (5%)
Urothelial cancer	20 (3%)	5 (4%)
Gastric cancer	20 (3%)	1 (1%)
Cervical cancer	18 (3%)	1 (1%)
Others	17 (3%)	1 (1%)
Cancer of unknown primary origin (CUP)	16 (3%)	4 (4%)
Sarcoma	14 (2%)	3 (3%)
Head and neck cancer	14 (2%)	2 (2%)
Neuroendocrine cancer	13 (2%)	1 (1%)
Malignant Mesothelioma	12 (2%)	7 (6%)
Melanoma	12 (2%)	2 (2%)
Esophageal cancer	11 (2%)	2 (2%)
SCLC	11 (2%)	0
Hepatocellular cancer	10 (2%)	2 (2%)
Adrenocortical cancer	8 (1%)	0
Endometrial cancer	8 (1%)	1 (1%)
Thymoma	8 (1%)	1 (1%)
Renal cell carcinoma	6 (1%)	2 (2%)
Adenoid cystic carcinoma (salivary gland)	5 (1%)	1 (1%)
Myoepithelial carcinoma	4 (0.5%)	0
Glioblastoma	4 (0.5%)	0
Anogenital cancer	3 (0.5%)	0
Germ cell cancer	2 (0.5%)	0
Vulvovaginal cancer	2 (0.5%)	1 (1%)

^a^Basal cell carcinoma is not included

**Fig. 1:**
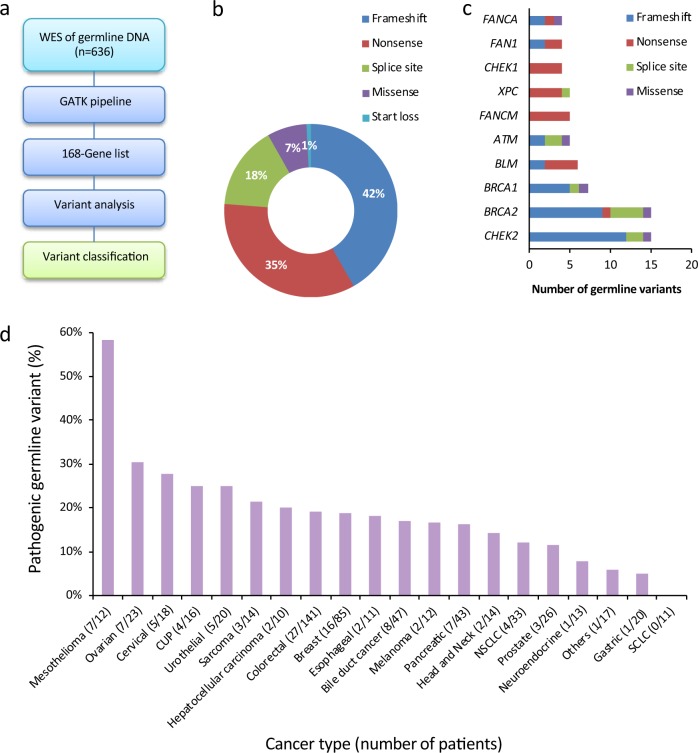
Identification and classification of pathogenic germline variants. **a** Schematic outline of the strategy for identification of pathogenic germline variants in the cohort. In total, whole-exome sequencing (WES) data from 636 patients were used for variant calling with GATK bioinformatic pipeline, followed by filtering using a prespecified gene list (Supplementary Table 1). Variant analysis and classification was done as described in the Methods section. **b** Distribution of mutational type among the 121 identified pathogenic and likely pathogenic mutations was calculated. **c** The most commonly mutated genes (>three pathogenic or likely pathogenic variants) are shown, ranked from bottom to top with *CHEK2* and *BRCA2* being the most frequently mutated of the genes included in this study (both *n* = 15). Distribution of mutational type is included in the figure. **d** The fraction of pathogenic or likely pathogenic variant was calculated for each cancer type and the graph shows the ranking of cancer types with highest fraction of germline variant. Only cancer types represented by >10 patients are included in the Figure. The bracket after each cancer type indicates the number of patients with germline variant/total number of patients for each cancer type

In total, 121 germline variants classified as pathogenic (*n* = 110) or likely pathogenic (*n* = 11) were identified in only 42 out of the 168 investigated genes (25%). Variants consisted of 51 frameshift variants (42%), 42 nonsense variants (35%), 18 splice-site variants (15%), 9 missense variants (7%) and one start loss variant (1%) (Fig. 1b, Supplementary Table 4). A small number of genes were overrepresented with respect to germline mutational burden. Fifteen pathogenic variants were identified in *BRCA2* and *CHEK2*, while seven pathogenic variants were identified in *BRCA1*, thus, about one third of the pathogenic variants were identified in these three genes (Fig. 1c, Supplementary S4). The *CHEK2* variant c.1100del, p.Thr367Metfs*15, which has an allele frequency of 0.2% in non-Finish Europeans (GnomAD) and is known to have reduced penetrance^38^ was e.g., observed in 12 patients (1.9%). Most variants were however only represented 1–3 times, thus excluding any founder effect on the results. Aside from the 10 genes that were most frequently found to have germline pathogenic variants, no genes were found to have such variants in more than three individuals (Fig. 1c, Supplementary Table 4).

Pathogenic variants were associated with every tumor type found in at least 10 individuals, except in the case of patients suffering from small cell lung cancer (SCLC) (Fig. 1d). However, the variant frequency varied greatly over the different tumor types. The incidence of patients with germline pathogenic variants ranged from 58% in malignant mesothelioma, 30% in ovarian cancer, 28% in cervical cancer, 25% in cancer of unknown primary origin and 25% in urothelial cancer, and down to 5% in gastric cancer (Fig. 1d).

### Pathogenic variants affecting HR

**Fig. 2:**
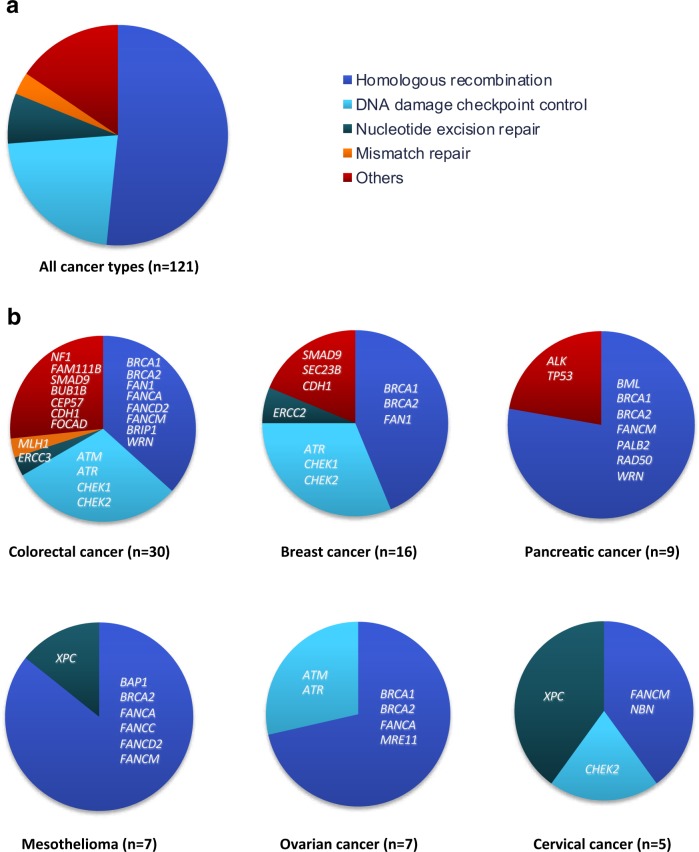
Germline variants are predominantly found in genes involved in DNA checkpoint and repair pathways. **a** Distribution of molecular pathways among the 121 identified pathogenic or likely pathogenic variants. **b** For each of the selected cancer types; colorectal cancer, breast cancer, pancreatic cancer, mesothelioma, ovarian cancer, and cervical cancer, the distribution of molecular pathways are shown. The mutated genes are indicated in the figure. Full information about each variant is found in Supplementary Table 4. The number of patients for each cancer type is shown in bracket

Genes encoding proteins within the HR pathways, including *BRCA1*, *BRCA2*, and genes in the Fanconi anemia pathway, generally harbored more pathogenic germline variants (52%) than genes within other pathways such as DNA damage checkpoint control (22%), nucleotide excision repair (7%), MMR (3%) and other pathways (16%) (Fig. 2a). However, looking into the different tumor types, particularly pancreatic cancer, mesothelioma and ovarian cancer show a tendency for enrichment of pathogenic variants in genes within the HR pathway with 70–85% of the identified variants located within these genes; although the number of included patients within these cancer types is limited (Fig. 2b). Interestingly, more than half of the possible pathogenic variants identified in patients with mesothelioma were located within genes of the Fanconi anemia pathway (*FANCA*, *FANCC*, *FANCD2*, and *FANCM*). Furthermore, remarkably, only a single pathogenic variant (*MLH1* p.Pro648Ser) within the MMR genes was identified in patients with CRC (Fig. 2b, Supplementary Table 4).

### Loss of Heterozygosity (LOH) and second hit

**Fig. 3:**
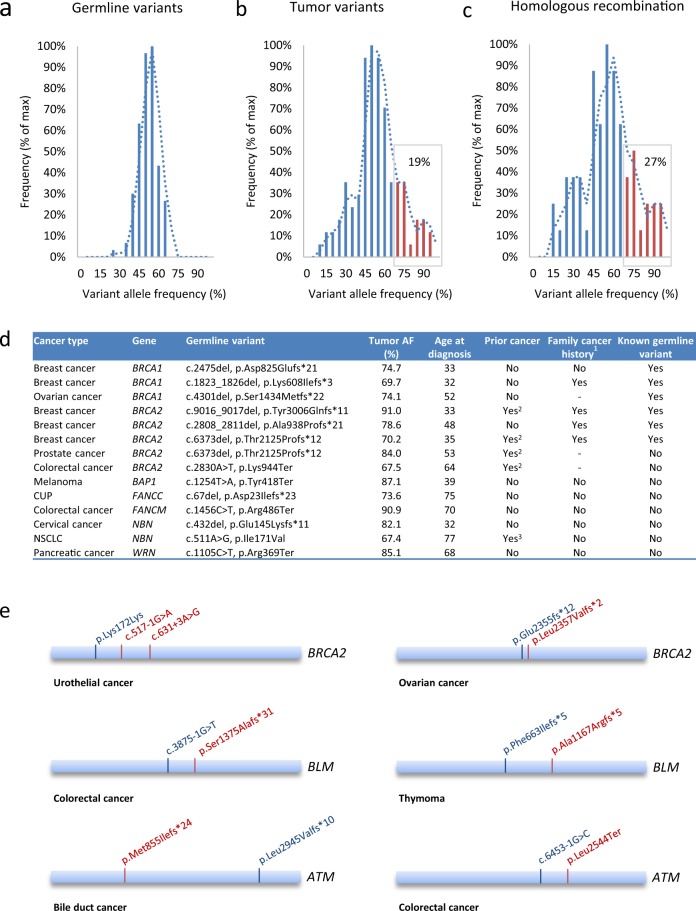
Analysis of loss-of-heterozygosity (LOH) and second hits from whole-exome-sequencing data from paired tumor samples. **a** The histogram shows variant allele frequency distribution of all germline variants (*n* = 121), demonstrating that most variants are called close to 50% as expected for heterozygous variants. **b** Histogram of tumor allele frequencies of all variants shown in **a**. **c** The histogram shows tumor allele frequencies of the variants in homologous recombination genes. Variants with tumor allele frequency > 65% are indicated in red. **d** An overview of patients with variants in homologous recombination pathway genes with allele frequency > 65%. **e** Potential second hits are shown; germline variants are indicated in blue, while somatic variants are shown in red. ^1^Known cancer in first or second degree relatives, ^2^Breast cancer, ^3^Melanoma

To further elucidate the causative role of the identified pathogenic germline variants, the individual tumor samples were assessed for the allele frequency of the identified germline variants, as loss of heterozygosity (LOH), thus, loss of the wild-type allele, can indicate causality. However, it is important to state that LOH can also occur by chance as the consequence of another pathogenic variant, and that causality of a pathogenic variant cannot be excluded solely based on missing LOH and vice versa. The allele frequency of all identified germline variants in blood samples was <65%, indicating that only heterozygous variants were identified (Fig. 3a). When analyzing each of the pathogenic germline variants in the tumor sample from the same patient, allele frequencies above 65% were observed, indicating LOH of these genomic regions. Due to the fact, that the tumor biopsies contained varying amounts of normal tissue, LOH may be under estimated using this approach. Furthermore, tumor heterogeneity may mask LOH in a subpopulation of cancer cells. On the contrary, chromosomal instability may give rise to LOH of genes that are not causal for the cancer, showing the results should be interpreted with caution. Using the cut-off value of 65% allele frequency, 19% of the overall identified pathogenic germline variants showed LOH in tumor samples (Fig. 3b), while 27% of the identified germline variants known to play a role in HR showed LOH in tumor samples (Fig. 3c), thus, supporting the importance of HR in the development of cancer in this patient cohort. The tumor allele frequency of all identified pathogenic germline variants are indicated in Supplementary Table 4. Focusing on the germline variants identified within genes involved in HR, most variants showing LOH were in agreement with known genotype–phenotype correlation as indicted by the observation of *BRCA1/*2 variants in breast, ovarian and prostate tumor samples and *BAP1* variants in uveal melanoma (Fig. 3d). However, also less well-established correlations were identified. For example, a *BRCA2* nonsense variant (p.Lys944Ter)^39^ and a *FANCM* nonsense variant (p.Arg486Ter)^40^ displayed LOH in the tumor samples of two patient with CRC, as well as LOH of a *NBN* missense variant (p.Ile171Val) in the tumor sample of a patient with NSCLC previously suggested to increase the risk of developing NSCLC.^41^ In addition, a *WRN* nonsense variant (p.Arg369Ter) showed a high allele frequency (>85%) in the tumor sample of a patient with pancreatic cancer in line with previous suggestions that loss-of-function variants within *WRN* might be associated with an increased predisposition to pancreatic cancer.^42^ Of note, the LOH in tumor tissue detected by NGS was confirmed by SNParray.

Another indication of causality of germline variants is, according to Knudson’s two-hit hypothesis,^43^ the identification of a second hit in the tumor sample within the same gene as the identified germline variant but on the opposite allele. In total, we identified a pathogenic second hit in six patients (Fig. 3e). Some of these findings, such as second hits in *ATM* and *BLM* in CRC patients, were supportive of previously suggested cancer associations.^44,45^ A second hit in *BRCA2* was also observed in a patient with ovarian cancer, however, further analysis revealed that the germline variant, c.7069_7070del, p.Leu2357Valfs*2, and the somatic variant, c.7065del, p.Glu2355Aspfs*12, were located on the same allele, thus, the somatic variant would be expected to restore the interrupted reading frame (resulting in p.Glu2355_Phe2356delinsAsp) reversing the pathogenicity of the germline variant. This paradoxical phenomenon of a restoring mechanism has previously been described for *BRCA* variants in platinum resistant ovarian cancer.^46,47^

We also identified previous unknown associations such as a second hit in *ATM* of a patient with bile duct cancer and a second hit in *BLM* in a patient with thymoma. Furthermore, a patient with urothelial cancer and a pathogenic germline variant in *BRCA2* was found to have two second hits. The two somatic variants, c.517-1G>A and c.631+3A>G, were located on opposite alleles, however, it was not possible to determine the allelic location of the germline variants in the other cases (Fig. 3e).

### Co-occurrence of germline and somatic variants in the HR pathway

**Fig. 4:**
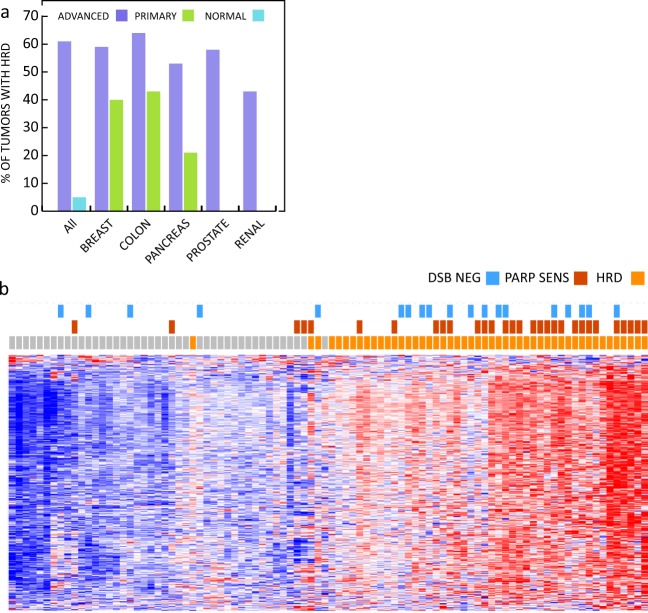
Homologous recombination defects in tumors from patients with advanced cancer. Panel **a** shows a comparison of the percentage of tumors with HRD in primary cancers from breast, colon, pancreas, prostate and kidney compared to tumors from patients with advanced cancer. Panel **b** depicts a two-way hierarchical cluster of tumors from patients with inactivating germ-line mutations in genes encoding proteins involved in double strand break repair with (blue) or without LOH or somatic mutations (no label) by their HRD status. Signatures for HRD and PARP sensitivity were derived from previously.^48^ Briefly, the 534 tumors where expression arrays were available were clustered according to the reported gene lists before an KNN based classifier was generated. All samples were subsequently classified as HRD positive/negative or PARP inhibitor sensitive/insensitive with a predictive value for HRD of 96% and for PARP sensitivity 95%, respectively. Tumors with deficient HR repair are depicted in orange and tumors with normal HR repair are labelled in gray, respectively. Predicted PARP inhibitor sensitive tumors are labelled in red

Furthermore, we examined the occurrence of somatic pathogenic variants in genes associated with HR repair and DSB repair and characterized the gene expression profiles of 534 tumors from the patients. Ninety-two of the tumors were from patients with germline variants. In total, 57 tumors exhibited mutations in the HR pathway and more than half of these mutations were found in *BAP1* and *BRCA2*. Seven of the mutations occurred in tumors from patients carrying pathogenic germline variants. Employing previously established gene expression signatures for HR deficiency (HRD) and PARPi sensitivity,^48^ we noticed that tumors from patients with advanced cancer exhibit a higher degree of HRD than primary cancers (Fig. 4a). Normal tissues do not exhibit HRD with the exception of testis, which was included as control. 79% (15 out of 19) of the tumors with LOH or a concurrent somatic mutation were predicted to have HRD and 37% (7) of these were predicted to be PARPi sensitive (Fig. 4b). Taken together, we infer that co-occurrence of germline and somatic variants in the HR pathway may play a role in cancer development and treatment response.

### BRCA in atypical cancer types and less established associations in breast cancer

Overall, pathogenic *BRCA1* and *BRCA2* germline variants were observed in patients with expected malignancies such as breast, ovarian, prostate and pancreatic cancer, a large proportion of which showed loss of the wild-type allele in the tumor sample (Fig. 3d). *BRCA1/2* germline variants were also observed in patients with other more atypical cancer types such as salivary adenoid cystic carcinoma, bile duct cancer, endometrial cancer, mesothelioma, esophageal cancer and urothelial cancer, however, without a clear loss of the wild-type allele in the tumor sample (Supplementary Table 4). Pathogenic *BRCA1* and *BRCA2* variants were identified in 7% of the investigated patients with breast cancer. In addition, this patient group harbored pathogenic germline variants in other genes previously known or suggested to be associated with breast cancer to a varying extent, including *ATR*, *CDH1*, *CHEK1*, *CHEK2*, *ERCC2*, *FAN1*, *SEC23B*, and *SMAD9*. However, with the exception of a single patient carrying the moderately penetrant *CHEK2* variant, c.1100del, p.Thr367Metfs*15, none of these variants had indications of LOH in the breast tumor samples.

### Categorization of variants in respect to clinical significance

To further evaluate whether variants played a role in the cancer etiology of each case, all pathogenic variants were categorized into three groups based on their clinical significance and thus potential causality (Supplementary Table 4). Group 1 includes likely causal variants, representing genes known to be associated with an increased risk of the given cancer type, e.g. *BRCA1 and BRCA2* in breast, ovarian and pancreatic cancer, *BAP1* in melanoma and mesothelioma, etc. This group also includes variants with a reduced penetrance, e.g., the *CHEK2* c.1100del variant in breast cancer. About one fourth of the variants falls into this category and are thus likely to be causative and a primary diagnostic finding relevant to the cancer type. Group 2, constituting about 20% of the variants, represents interesting findings that show new possible associations between a gene and a given cancer type but with limited or no previously known predisposition. This group also includes variants with a high tumor allele frequency or genes/gene-families overrepresented in a given cancer type in our data set, e.g. genes in the Fanconi anemia pathway observed in mesothelioma. Group 3, representing about half of the cases, includes genes known to be associated to a cancer predisposition syndrome but unlikely to be causative for the observed phenotype, e.g. based on lacking expression of the variant in the tumor sample, or where lack of evidence from the literature in a given cancer type makes the causality inconclusive.

It should be noted, however, that these categorizations are merely an assumption of causality. A strong family history and clear co-segregation data would provide further evidence for causality. However, due to ethical considerations and the scope of this study it was not possible to obtain pedigrees or blood samples of relatives in each investigated family.

### Clinical implications for detection of pathogenic germline variants

**Fig. 5:**
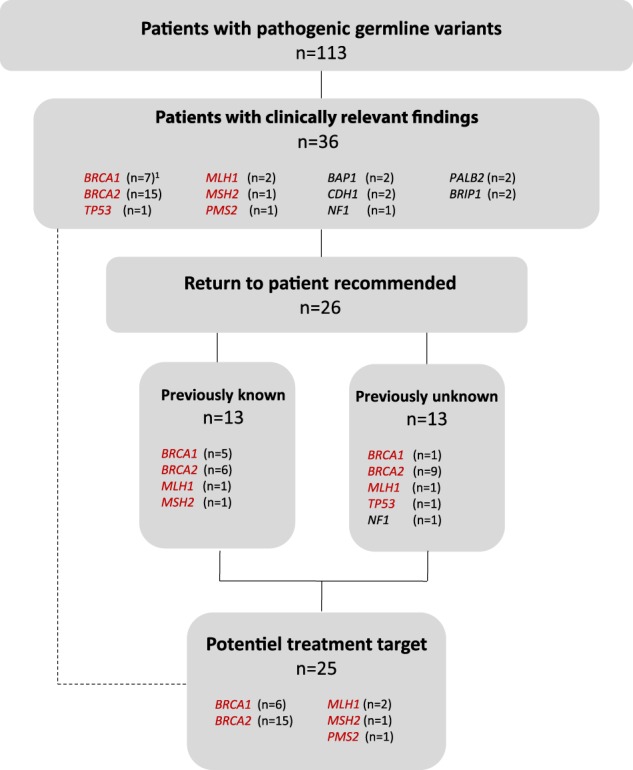
Flowchart highlighting the clinical utility of germline testing in our cohort. Pathogenic germline variants were found in 113 patients. A relevant treatment could have been recommended in 25 patients based on the identified variant. Thirty-six variants were selected for further evaluation due to potential clinical implication for the patients or their families (Supplementary Table 5). In 26 cases, return was recommended based on ACMG/AMP recommendations, recent evidence and the individual family history. Of these, 13 variants were previously identified. ^1^No return was recommended for the *BRCA1* variant p.Arg1699Gln, since it is a moderate risk variant. Genes shown in red indicate inclusion in ACMG/AMP recommendations for return of results

Of the 113 patients carrying a pathogenic germline variant, the genetic findings were suggested to have a potential important implication to 36 of the patients and/or their families with respect to either treatment, prophylactic surgery, inclusion in screening programs or genetic counseling (Fig. 5; Supplementary Table 5). Since this study was done retrospectively, the majority of the patients were deceased and, in these cases, return of the results had no clinical relevance, neither in respect to treatment of the patients nor from a diagnostic point of view. Due to ethical considerations, we have chosen to follow the ACMG/AMP recommendations, in combination with recent evidence and the individual family history. In total, 26 variants were recommended returned to the patients or their families. Half of these patients had no previous implications of hereditary cancer predisposition and variants had not previously been identified in their families. Potential actionable treatment targets were identified in 25 patients (4%) and included PARPi for patients with a pathogenic *BRCA1/2* variant (*n* = 21) and immunotherapy for patients harboring a pathogenic variant within the MMR genes (*n* = 4).

## Discussion

Predisposing germline variants has long been recognized for specific cancer types, such as breast, ovarian and CRC. Genomic screening is mainly based on the age at onset and family history, whereas it is unclear if predisposing germline variants are overlooked in other patient populations. Current results from germline studies of The Cancer Genome Atlas^49^ or datasets derived from routine clinical tumor sequencing for allocation of cancer patients to early clinical trials^8–11^ have indicated that variants in cancer susceptibility genes may be more frequent in atypical cancer forms than anticipated.

We analyzed the presence of germline variants in consecutive patients with advanced cancer from the CoPPO study that comprise patients with exhausted treatment options and observed a prevalence of pathogenic germline variants in ~18% among the patients.

As mentioned, studies have reported pathogenic germline variants in various cancer populations.^8–11,49^ Our data are remarkably similar to the recent results from Mandelker et al. that discovered pathogenic variants in 19.7% of advanced cancer patients.^8^ While we employed a gene list of 168 genes previously associated with cancer, Mandelker et al. employed a gene panel of 76 genes, all of which were included in our study, except for five genes where no pathogenic variants were identified. Pathogenic variants were identified in only 25% of the 168 genes investigated in our study, indicating that germline testing may be feasible with a relatively narrow list of genes. Generally, there is a high consistency in the frequency of pathogenic variants in the overlapping genes; although Mandelker et al. reports a larger number of variants within *APC* and the MMR genes. Of notice, the gene panel employed by Mandelker et al. did not include any of the genes in the Fanconi anemia pathway, where we observed a relatively large proportion of pathogenic variants, while our study did not include heterozygous variants in *MUTYH* in the overall frequency of identified germline variants in contrast to Mandelker et al. Our cohort shares similarities with the study from Mandelker et al. comprising advanced cancer patients and being rich in breast and CRC. In contrast, cohorts from other studies vary both in the number of cancer types and the composition of advanced versus primary cancers.^45,50–53^

Mesothelioma exhibited the highest frequency of pathogenic germline variants with seven out of twelve patients (58%) being carriers. Germline variants in mesothelioma have previously been reported with a frequency of 10–12% in patients.^49,51^ In concordance with previous studies, the majority of the mutated genes were involved in HR (*BAP1*, *BRCA2*, *FANCA*, *FANCC*, *FANCD2*, and *FANCM*) and a single gene was involved in nucleotide excision repair (*XPC*). *BAP1* is the best studied predisposition gene for mesothelioma, supported by both clinical findings and mouse models.^54^ However, our results may suggest a more heterogeneous genetic background for the disorder, dominated by genes involved in HR. Three of the mesothelioma patients carrying a pathogenic germline variant (in *FANCD2*, *FANCM*, and *XPC*) had first-degree relatives with mesothelioma and a known history of asbestos exposure, implying a gene-environment interaction, where the genetic background increases the sensitivity of the patient to the carcinogenic effect of asbestos.^55^ The high frequency of pathogenic germline variants in mesothelioma indicates that this group of patients should be offered mutation screening on a routine basis—especially if they have a family history of mesothelioma or other cancer types associated with mutations in HR genes. Furthermore, in the light of the promising effects of PARPi therapy in treatment of *BRCA* positive ovarian and breast cancer patients, clinical trials on PARPi treatment in patients with mesothelioma could be warranted.

CRC was the predominant cancer type and 19% of the patients exhibited a pathogenic germline variant. Among these only a single variant was found in one of the four MMR genes that predispose to HNPCC.^53,56^ Neither did we identify pathogenic variants in the *APC* gene that predispose to FAP, nor did we observe homozygous or compound heterozygous germline variants in the recessive CRC predisposition genes *MUTYH* and *NTHL1*. This may be related to the fact that most Danish HNPCC and polyposis families are under surveillance by the national registry as Denmark has had a long tradition for registration and screening of HNPCC families. Consequently, the majority of HNPCC families are now included in the relevant screening programs and subject to preventive treatment. Therefore, few patients are expected to develop advanced cancer due to the successful preventive program. Obviously, patients with *de novo* MMR mutations could be represented in our study, but a number of studies have indicated that Lynch de novo mutation are relatively rare compared to e.g., *APC* related polyposis and it is estimated that only 1–3% of all HNPCC cases are caused by MMR de novo mutations.^57^

Due to the clinical setting of the CoPPO trial, cancers in our study, in general, represent a heavily pretreated, drug-resistant phenotype with a median of three prior standard treatment regimens (range 1–12).^5^ We speculate that the selection of patients in our cohort with advanced cancer displaying a drug-resistant phenotype might be enriched for either germline or somatic variants within specific pathways such as the HR pathway. Paradoxically, HRD is known to predict good response to platinum-based chemotherapy,^32^ however, the majority of patients in our cohort have already previously been treated with chemotherapy such as cisplatin, carboplatin or oxaliplatin, either alone or in combination with other chemotherapy. Therefore, even though we report findings of previously undiscovered pathogenic germline variants, particularly in *BRCA1* and *BRCA2*, the potential clinical benefit of PARPi treatment in these drug-resistant patients at advanced stage of cancer remains unclear.

The occurrence of pathogenic germline variants in patients with advanced cancer raises the question of whether genetic testing should become part of the clinical routine for a group of patients. In this context it is important to distinguish between pathogenic variations in the well-established actionable genes such as *BRCA1* and *BRCA2* and novel candidate genes such as *FANCM* and *BLM*. Of note, we could classify about haft of the variants as having either likely or possible clinical significance for the cancer; however, with the caution that further validation is needed to confirm these potential causalities, including pedigree of affected families and functional studies of variants. Counseling is generally based on solid cohort and co-segregation data, but these may not be available for variants in the emerging group of cancer susceptibility genes. Obviously, we could decide to selectively screen a small panel of actionable cancer genes, but we risk that patients or healthy carriers exhibiting pathogenic variants in emerging factors may wrongly be excluded from targeted treatment or pre-symptomatic screening programs. Another direction is to employ structural and functional analysis for variant classification. In this context recent procedures to establish defects in HR from mutation patterns may pave the way for including emerging genes and variants in genetic testing and counseling.^58,59^ Evidently, unfounded classification of genetic variants is harmful to the patient and great care should be taken to generate common protocols and collaborative efforts to meet the required standards.

In conclusion, our study of patients with advanced solid cancers showed a high frequency of germline variants, especially in the HR pathway. This implies that testing for selected germline variants in precision oncology may contribute to the improved treatment of cancer patients.

## Methods

### Patients

This study included a cohort of patients enrolled in the Copenhagen Prospective Personalized Oncology (CoPPO) study (NCT02290522) from May 2013–February 2018.^5,60^ The CoPPO study aims to investigate the clinical utility of using molecular profiling to select patients for phase 1 trials. Patients with exhausted treatment options considered eligible for phase 1 trials were offered enrolment. All patients fulfilled the inclusion criteria including: life expectancy ≥ 3 months, normal organ function, age ≥ 18 years, Eastern Cooperative Oncology Group (ECOG) performance status 0 or 1, and lesions assessable for biopsy. Basic characteristics and clinical information were collected at baseline in a prospectively collected database. Regulatory approvals from the Regional Ethics Committee and the Danish Data Protection Agency were obtained (Danish Ethical Committee, file number: 1300530). All patients provided signed informed written consent.

### Whole exome sequencing

Whole exome sequencing (WES) was performed as previously described.^5^ In brief, DNA was extracted from whole blood samples and matched tumor biopsies. Sequencing libraries were prepared from 200 ng of DNA. Fragmentation was done on Covaris S2 (Agilent) to ~300 base pair fragments and adaptor ligation was performed using KAPA HTP Library Preparation Kit. Exomes were enriched with SureSelectXT Clinical Research Exome kit (Agilent). Paired-end sequencing was performed, aiming at an average coverage of 50–100×, using the HiSeq2500 or NextSeq500 platforms from Illumina.

### Sequencing data pre-processing

Sequenced reads were trimmed and mapped to hg19/GRCh37 reference genome using BWA-MEM v0.7.12 software.^61^ Alignment quality control was performed with mosdepth v0.2.0^62^ for all the exons of the genes of interest (Supplementary Table 1). Alignment files were pre-processed with GATK v3.8.0 suite^63^ using Best Practices guidelines. For germline variant calling alignment files were analyzed with GATK v3.8.0 suite’s HaplotypeCaller^64^ using Best Practices guidelines for germline variant calling. Variant files were filtered so all the variants: (1) were covered by at least 10 reads, (2) had variant score higher or equal to 20, (3) were within the genes of interest using a list of 168 cancer-associated genes (Supplementary Table 1). The list was based on cancer-related genes from the ACMG/AMP’s recommendations, high penetrance cancer-related genes, and review of the literature for additional cancer-related genes with modes of inheritance.^6,7,10,11^ Somatic variant calling in tumor samples was performed by MuTect2^39^ from GATK v3.8.0 suite.

### Variant analysis

Called germline variants were filtered using Ingenuity Variant Analysis (IVA; http://ingenuity.com). First, variants with call quality < 20 and read depth < 10; were disregarded. Second, variants with an allele frequency > 1% of the public variant database including 1000 genomes project (http://www.1000genomes.org), ExAC (http://exac.broadinstitute.org), GnomAD (http://gnomad.broadinstitute.org) or NHLBI ESP exomes (http://evs.gs.washington.edu/EVS/), or unless established as a pathogenic common variant, were excluded. Third, only coding non-synonymous variants and splice-site variants (+/−2bp) were kept. Finally, only variants with an allele frequency > 20% were kept for further analysis.

### Variant classification

All variants identified after IVA processing were divided into subgroups based on the classification provided by IVA. All *pathogenic* or *likely pathogenic* variants were manually classified following ACMG/AMP’s recommendations,^40^ thus, using information about population allele frequency, in silico tools including MaxEntScan^65^ for splice-site variants and Align-GVGD^66^ for missense variants, literature search and in-house databases. Furthermore, variants within *BRCA1* and *BRCA2* and within the MMR genes (*MLH1*, *MSH2*, *MSH6*, and *PMS2*) classified as *uncertain* by IVA were manually classified using the same procedure as well as the guidelines provided by ENIGMA and InSiGHT, respectively. Finally, all frameshift and nonsense variants classified as *uncertain* were manually curated following the same procedure. All variants classified as pathogenic (Class 5) or likely pathogenic (Class 4) after manual curation were considered for further analysis, while variants classified as of unknown significance (Class 3), likely benign (Class 2) or benign (Class 1) were disregarded.

### Transcriptome based analysis of HRD and sensitivity to cisplatin and PARP inhibitors

Signatures of HR repair and DSB repair were examined in a set of primary tumors^67^ and in 534 tumors from the patients in this study; 92 of these tumors were from patients with pathogenic germline variants. Briefly, total RNA was isolated and processed as described^68^ before samples were labeled according to the manufacturer’s guidelines (Affymetrix, Santa Clara, CA, USA). The labeled cRNAs were hybridized to the HG-U133plus2 GeneChip array, which query close to 48,000 genes by ∼56,000 probe sets. After scanning, the data were RMA normalized and imported in Qlucore Omics Explorer for further analysis. The classification was based on gene expression signatures for HR deficiency, cisplatin and PARPi sensitivity derived from McGrail et al.^48^ These signatures were originally generated from expression data of 857 solid tumor cell lines from the Cancer Cell Line Encyclopedia (CCLE) matched with drug sensitivity data from the Genomics of Drug Sensitivity in Cancer (GDSC). Gene lists of each signature were initially employed to provide a two-way hierarchical cluster of the advanced cancer samples before two groups of characteristic samples were used to build a second K-means based classifier that could be employed in Qlucore Omics Explorer.

### Ethical considerations regarding return of results

At study enrollment, patients received information about potential germline findings and a written consent with degree of return of germline findings was signed. The consent included a remark stating the obligation to contact the patient or family members with important information concerning specific germline findings. Regarding results from this retrospective study, all pathogenic germline variants with potential clinical implications were evaluated and return of results was recommended based on ACMG/AMP’s recommendations,^6,7^ recent evidence and the individual family history. For all variants subjected to return, genetic counselling of the patient and validation of results using an independent blood sample were recommended. Furthermore, these variants will be submitted to ClinVar.

### Reporting summary

Further information on experimental design is available in the Nature Research Reporting Summary linked to this paper.

## Supplementary information

Supplementary Material

Reporting Summary Checklist

## Data Availability

The authors confirm that all relevant data are included in the paper and its supplementary information files. Raw DNA sequencing data (fastq and unfiltered vcf files) generated during and/or analyzed during hte current study are not publically available due to consideration of current data protection regulations as well as limitations in the written informed consent signed by the patients. These data are available from the corresponding author upon reasonable request. Filtered and validated variants will be available in ClinVar. Microarray data are uploaded to Gene Expression Omnibus (GEO) with accession number GSE131027.

## Supplementary information

**Supplementary information** accompanies the paper on the *npj Genomic Medicine* website (10.1038/s41525-019-0087-6).
