# Supplementary material for: High frequency of pathogenic germline variants within homologous recombination repair in patients with advanced cancer
Source: NPJ Genom Med. 2019 Jun 21;4:13. doi: 10.1038/s41525-019-0087-6 (PMC6588611; doi:10.1038/s41525-019-0087-6)
Supplement: Supplementary file 1 — Supplementary Material [file 41525_2019_87_MOESM1_ESM.pdf]

SUPPLEMENTARY INFORMATION

Supplementary Table 1. **Gene list used for germline analysis.** The genes of interest comprise 168 cancer-associated genes, based on cancer-related genes from the ACMG/AMP's recommendations, high penetrance cancer-related genes, and review of the literature for additional cancer-related genes with modes of inheritance.

Supplementary Table 2. **Patients with more than one pathogenic variant.** Six cases carried 2 or 3 pathogenic variants.

Supplementary Table 3. Patients with *MUTYH* or *NTHL1* variants. Cases with heterozygote *MUTYH* or *NTHL1* variants were excluded from further analysis.

Supplementary Table 4. **List of all 121 identified pathogenic and likely pathogenic variants.** Cancer type for patient with germline variant is shown. For missense variants Align-GVGD was used for *in silico* analysis. Overall allele frequencies according to The Genome Aggregation Database (GnomAD_All) and non-Finish Europeans (GnomAD_NFE) are shown together with tumor allele frequencies (Tumor_AF).

Supplementary Table 5. Patients (n=36) were selected for further evaluation. In 26 cases, return was recommended based on ACMG/AMP recommendations, recent evidence and the individual family history.

Supplementary Table 1. Gene list used for germline analysis.

Gene	NM number
ABRAXAS1	NM_139076
ACD	NM_022914
AIP	NM_003977
AKT1	NM_005163
ALK	NM_004304
APC	NM_000038
ARNT2	NM_014862
ATM	NM_000051
ATR	NM_001184
AXIN2	NM_004655
BAP1	NM_004656
BARD1	NM_000465
BLM	NM_000057
BMPR1A	NM_004329
BRCA1	NM_007294
BRCA2	NM_000059
BRIP1	NM_032043
BUB1B	NM_001211
CBL	NM_005188
CDC73	NM_024529
CDH1	NM_004360
CDK4	NM_000075
CDKN1A	NM_001291549
CDKN1B	NM_004064
CDKN1C	NM_000076
CDKN2A p16INK4a	NM_000077
CDKN2A p14ARF	NM_058195
CDKN2B	NM_004936
CDKN2C	NM_001262
CEBPA	NM_004364
CEP57	NM_014679
CHEK1	NM_001330427
CHEK2	NM_007194
COL1A1	NM_000088
COL3A1	NM_000090
COL5A1	NM_000093
COL5A2	NM_000393
CTNNA1	NM_001903
CTR9	NM_014633
CYLD	NM_015247
DDB2	NM_000107
DICER1	NM_177438
DIS3L2	NM_152383
DKC1	NM_001363
EGFR	NM_005228
EGLN1	NM_022051
EGLN2	NM_053046

EPAS1	NM_001430
EPCAM	NM_002354
ERCC2	NM_000400
ERCC3	NM_000122
ERCC4	NM_005236
ERCC5	NM_000123
ETV6	NM_001987
EXT1	NM_000127
EXT2	NM_207122
EZH2	NM_004456
FAM111B	NM_198947
FAN1	NM_014967
FANCA	NM_000135
FANCB	NM_152633
FANCC	NM_000136
FANCD2	NM_033084
FANCE	NM_021922
FANCF	NM_022725
FANCG	NM_004629
FANCI	NM_001113378
FANCL	NM_018062
FANCM	NM_020937
FH	NM_000143
FLCN	NM_144977
FOCAD	NM_017794
GALNT12	NM_024642
GATA2	NM_001145661
GNAS	NM_000516
GPC3	NM_004484
GREM1	NM_013372
HNF1A	NM_000545
HNF1B	NM_000458
HOXB13	NM_006361
HRAS	NM_005343
IDH1	NM_005896
INHA	NM_002191
INHBA	NM_002192
IPMK	NM_152230
KIF1B	NM_015074
KIT	NM_000222
MAX	NM_002382
MDH2	NM_005918
MEN1	NM_130799
MET	NM_000245
MITF	NM_198159
MLH1	NM_000249
MLH3	NM_001040108
MRE11A	NM_005591
MSH2	NM_000251
MSH6	NM_000179

MUTYH	NM_012222
NBN	NM_002485
NF1	NM_001042492
NF2	NM_000268
NSD1	NM_022455
NTHL1	NM_002528
PALB2	NM_024675
PAX5	NM_016734
PDGFRA	NM_006206
PHOX2B	NM_003924
PIK3CA	NM_006218
PMS1	NM_000534
PMS2	NM_000535
POLD1	NM_002691
POLE	NM_006231
POLH	NM_006502
POT1	NM_015450
PRF1	NM_001083116
PRKAR1A	NM_002734
PRSS1	NM_002769
PTCH1	NM_000264
PTEN	NM_000314
PTPN11	NM_002834
RAD50	NM_005732
RAD51	NM_133487
RAD51B	NM_133509
RAD51C	NM_058216
RAD51D	NM_002878
RB1	NM_000321
RBBP8	NM_002894
RECQL	NM_002907
RECQL4	NM_004260
RET	NM_020975
RHBDF2	NM_024599
RINT1	NM_021930
RPS20	NM_001146227
RUNX1	NM_001754
SBDS	NM_016038
SDHA	NM_004168
SDHAF2	NM_017841
SDHB	NM_003000
SDHC	NM_003001
SDHD	NM_003002
SH2D1A	NM_002351
SEC23B	NM_006363
SLX4	NM_032444
SMAD4	NM_005359
SMAD9	NM_001127217
SMARCA4	NM_001128849
SMARCB1	NM_003073

SPINK1	NM_003122
STK11	NM_000455
SUFU	NM_016169
TERF2IP	NM_018975
TERC	NR_001566
TERT	NM_198253
TGFBR1	NM_001306210
TINF2	NM_001099274
TMEM127	NM_017849
TP53	NM_000546
TSC1	NM_000368
TSC2	NM_000548
UBE2T	NM_014176
UIMC1	NM_001199297
VHL	NM_000551
WRN	NM_000553
WT1	NM_024426
XPA	NM_000380
XPC	NM_004628
XRCC2	NM_005431
XRCC3	NM_001100119

Supplementary Table 2. Patients with more than one pathogenic variant.

Case	Chr	Position	Gene	Nucleotide change	Protein change	Classification	Tumor_AF
**Case A - male with Colorectal cancer**	15	40494896	*BUB1B*	c.1734+1G>T		Class 4	66.3
	22	29091856	*CHEK2*	c.1100del	p.Thr367Metfs*15	Class 5	37.8
**Case B - female with Colorectal cancer**	15	91346807	*BLM*	c.3415C>T	p.Arg1139Ter	Class 5	23.3
	3	142274739	*ATR*	c.2320dup	p.Ile774Asnfs*3	Class 5	Variant not called
**Case C - male with Colorectal cancer**	3	10133885	*FANCD2*	c.3799del	p.Tyr1267Thrfs*15	Class 5	45.0
	11	58892376	*FAM111B*	c.816dup	p.Ala273Serfs*9	Class 5	Variant not called
**Case D - male with Urothelial cancer**	15	91328183	*BLM*	c.2695C>T	p.Arg899Ter	Class 5	No tumor sample
	3	37081786	*MLH1*	c.1667+2_1667+8delinsATTT		Class 5	No tumor sample
**Case E - male with Pancreatic cancer**	5	131951698	*RAD50*	c.3040C>T	p.Gln1014Ter	Class 5	32.7
	8	30938648	*WRN*	c.1105C>T	p.ArgR369Ter	Class 5	85.1
	2	30142996	*ALK*	c.530G>A	p.Trp177Ter	Class 5	41.8
**Case F - male with CUP**	13	32914569	*BRCA2*	c.6082_6086del	p.Glu2028Lysfs*19	Class 5	54.8
	22	29121230	*CHEK2*	c.444+1G>A		Class 4	44.4
	5	138266225	*CTNNA1*	c.2074C>T	p.Gln692Ter	Class 5	51.5

AF, allele frequency

Supplementary Table 3. Patients with *MUTYH* or *NTHL1* variants.

Cancer type	Chr	Position	Gene	Nucleotide change	Protein change	Predicted effect	Classification	Align-GVGD	GnomAD (%)	GnomAD_NFE (%)	Tumor_AF
Breast cancer	1	45797228	*MUTYH*	c.1178G>A	p.Gly393Asp	Missense	Class 5	C65	0.3	0.48	50.0
Breast cancer	1	45797228	*MUTYH*	c.1178G>A	p.Gly393Asp	Missense	Class 5	C65	0.3	0.48	Variant not called
Colorectal cancer	1	45797228	*MUTYH*	c.1178G>A	p.Gly393Asp	Missense	Class 5	C65	0.3	0.48	51.7
Colorectal cancer	1	45797228	*MUTYH*	c.1178G>A	p.Gly393Asp	Missense	Class 5	C65	0.3	0.48	77.4
NSCLC	1	45797228	*MUTYH*	c.1178G>A	p.Gly393Asp	Missense	Class 5	C65	0.3	0.48	56.6
SCLC	1	45797228	*MUTYH*	c.1178G>A	p.Gly393Asp	Missense	Class 5	C65	0.3	0.48	42.6
SCLC	1	45797228	*MUTYH*	c.1178G>A	p.Gly393Asp	Missense	Class 5	C65	0.3	0.48	Variant not called
Bile duct cancer	1	45798475	*MUTYH*	c.527A>G	p.Tyr176Cys	Missense	Class 5	C0	0.15	0.25	45.6
Bile duct cancer	1	45798475	*MUTYH*	c.527A>G	p.Tyr176Cys	Missense	Class 5	C0	0.15	0.25	4.0
CUP	1	45798475	*MUTYH*	c.527A>G	p.Tyr176Cys	Missense	Class 5	C0	0.15	0.25	25.1
NSCLC	1	45798475	*MUTYH*	c.527A>G	p.Tyr176Cys	Missense	Class 5	C0	0.15	0.25	47.8
Pancreatic cancer	1	45798475	*MUTYH*	c.527A>G	p.Tyr176Cys	Missense	Class 5	C0	0.15	0.25	64.4
Urothelial cancer	1	45798475	*MUTYH*	c.527A>G	p.Tyr176Cys	Missense	Class 5	C0	0.15	0.25	46.4
Pancreatic cancer	1	45798117	*MUTYH*	c.725G>A	p.Arg242His	Missense	Class 4	C0	0.0077	0.013	50.6
Colorectal cancer	16	2096239	*NTHL1*	c.268C>T	p.Gln90Ter	Nonsense	Class 5	-	0.14	0.20	41.9
Breast cancer	16	2096239	*NTHL1*	c.268C>T	p.Gln90Ter	Nonsense	Class 5	-	0.14	0.20	53.2
Breast cancer	16	2090005	*NTHL1*	c.859C>T	p.Gln287Ter	Nonsense	Class 5	-	0.016	0.021	46.6

CUP, Cancer of Unknown Primary
GnomAD, The Genome Aggregation Database
NFE, non-Finish Europeans
AF, allele frequency

Supplementary Table 4. **List of all 121 identified pathogenic and likely pathogenic variants.**

Cancer type	Chr	Position	Gene	Nucleotide change	Protein change	Predicted effect	Classification	Align-GVGD	GnomAD_all	GnomAD_NFE	Tumor AF	Clinical significance
Adenoid cystic carcinoma	17	41215947	*BRCA1*	c.5096G>A	p.Arg1699Gln	Missense	Class 5	C35	0.000024	0.000054	52.2	3
Bile duct cancer	11	108225581	*ATM*	c.8833_8834del	p.Leu2945Valfs*10	Frameshift	Class 5		0.000004	0.000009	43.8	2
Bile duct cancer	17	41226347	*BRCA1*	c.4675+1G>A		Splice site	Class 5				58.8	2
Bile duct cancer	13	32928997	*BRCA2*	c.7008-1G>A		Splice site	Class 5				52.1	2
Bile duct cancer	17	59853761	*BRIP1*	c.2097+1G>C		Splice site	Class 5				48.0	3
Bile duct cancer	3	10116274	*FANCD2*	c.2776C>T	p.Arg926Ter	Nonsense	Class 5		0.000024	0.000027	23.7	3
Bile duct cancer	16	23652477	*PALB2*	c.2T>C	p.Met1?	Start loss	Class 4		0.000004	0.000009	50.0	3
Bile duct cancer	12	21644525	*RECQL*	c.142C>T	p.Gln48Ter	Nonsense	Class 5		0.000008	0.000018	49.6	3
Bile duct cancer	5	236713	*SDHA*	c.1432_1432+1del		Splice site	Class 4		0.000004	0.000009	40.0	3
Breast cancer	3	142274739	*ATR*	c.2320dup	p.Ile774Asnfs*3	Frameshift	Class 5				Variant not called	3
Breast cancer	17	41245721	*BRCA1*	c.1823_1826del	p.Lys608Ilefs*3	Frameshift	Class 5		0.000004	0.000009	69.7	1
Breast cancer	17	41245072	*BRCA1*	c.2475del	p.Asp825Glufs*21	Frameshift	Class 5		0.000004	0.000009	74.7	1
Breast cancer	13	32911278	*BRCA2*	c.2787dup	p.Tyr930Ilefs*6	Frameshift	Class 5				60.8	1
Breast cancer	13	32911297	*BRCA2*	c.2808_2811del	p.Ala938Profs*21	Frameshift	Class 5		0.000012	0.000027	78.6	1
Breast cancer	13	32914859	*BRCA2*	c.6373del	p.Thr2125Profs*12	Frameshift	Class 5				70.2	1
Breast cancer	13	32953947	*BRCA2*	c.9016_9017del	p.Tyr3006Glnfs*11	Frameshift	Class 5		0.000004	0.000009	91.0	1
Breast cancer	16	68772227	*CDH1*	c.76G>T	p.Glu26Ter	Nonsense	Class 5				48.6	1
Breast cancer	11	125495891	*CHEK1*	c.236G>A	p.Trp79Ter	Nonsense	Class 5		0.001365	0.002290	29.2	3
Breast cancer	22	29091856	*CHEK2*	c.1100del	p.Thr367Metfs*15	Frameshift	Class 5		0.002077	0.002548	50.4	1
Breast cancer	22	29091856	*CHEK2*	c.1100del	p.Thr367Metfs*15	Frameshift	Class 5		0.002077	0.002548	72.6	1
Breast cancer	22	29091856	*CHEK2*	c.1100del	p.Thr367Metfs*15	Frameshift	Class 5		0.002077	0.002548	54.5	1
Breast cancer	19	45860755	*ERCC2*	c.1354C>T	p.Gln452Ter	Nonsense	Class 5				37.0	3
Breast cancer	15	31197786	*FAN1*	c.922_923del	p.Val308Cysfs*5	Frameshift	Class 5		0.000118	0.000251	57.3	2
Breast cancer	20	18523754	*SEC23B*	c.1603C>T	p.Arg535Ter	Nonsense	Class 5		0.000024	0.000036	43.0	2
Breast cancer	13	37439866	*SMAD9*	c.811C>T	p.Gln271Ter	Nonsense	Class 5				28.9	3
Cervical cancer	22	29091856	*CHEK2*	c.1100del	p.Thr367Metfs*15	Frameshift	Class 5		0.002077	0.002548	52.0	3
Cervical cancer	14	45658326	*FANCM*	c.5101C>T	p.Gln1701Ter	Nonsense	Class 5		0.001289	0.000996	47.7	3
Cervical cancer	8	90993009	*NBN*	c.432del	p.Glu145Lysfs*11	Frameshift	Class 5				82.1	2
Cervical cancer	3	14200140	*XPC*	c.1243C>T	p.Arg415Ter	Nonsense	Class 5		0.000012		Variant not called	3
Cervical cancer	3	14206926	*XPC*	c.779+2T>C		Splice site	Class 5				44.3	3
Colorectal cancer	11	108202280	*ATM*	c.7626_7629+1del		Splice site	Class 4		0.000004	0.000009	40.9	2
Colorectal cancer	3	142274739	*ATR*	c.2320dup	p.Ile774Asnfs*3	Frameshift	Class 5				Variant not called	3
Colorectal cancer	15	91346807	*BLM*	c.3415C>T	p.Arg1139Ter	Nonsense	Class 5		0.000008	0.000018	23.3	2
Colorectal cancer	15	91358377	*BLM*	c.4123del	p.Ser1375Alafs*31	Frameshift	Class 5				44.0	2
Colorectal cancer	13	32911322	*BRCA2*	c.2830A>T	p.Lys944Ter	Nonsense	Class 5		0.000008	0.000009	67.5	2
Colorectal cancer	17	59861629	*BRIP1*	c.1628+2T>G		Splice site	Class 5				29.0	3
Colorectal cancer	15	40494896	*BUB1B*	c.1734+1G>T		Splice site	Class 4				66.3	2
Colorectal cancer	16	68855903	*CDH1*	c.1712-1G>T		Splice site	Class 4				17.7	3
Colorectal cancer	11	95560975	*CEP57*	c.915_925dup	p.Leu309Profs*9	Frameshift	Class 5		0.000053	0.000018	49.2	3
Colorectal cancer	11	125495891	*CHEK1*	c.236G>A	p.Trp79Ter	Nonsense	Class 5		0.001365	0.002290	No tumor sample	3
Colorectal cancer	11	125495891	*CHEK1*	c.236G>A	p.Trp79Ter	Nonsense	Class 5		0.001365	0.002290	42.1	3
Colorectal cancer	22	29091856	*CHEK2*	c.1100del	p.Thr367Metfs*15	Frameshift	Class 5		0.002077	0.002548	54.6	1
Colorectal cancer	22	29091856	*CHEK2*	c.1100del	p.Thr367Metfs*15	Frameshift	Class 5		0.002077	0.002548	55.7	1
Colorectal cancer	22	29091856	*CHEK2*	c.1100del	p.Thr367Metfs*15	Frameshift	Class 5		0.002077	0.002548	48.5	1
Colorectal cancer	22	29091856	*CHEK2*	c.1100del	p.Thr367Metfs*15	Frameshift	Class 5		0.002077	0.002548	37.8	1
Colorectal cancer	22	29090054	*CHEK2*	c.1427C>T	p.Thr476Met	Missense	Class 4	C15	0.000340	0.000579	80.7	1
Colorectal cancer	2	128018833	*ERCC3*	c.2034dup	p.Phe679Ilefs*8	Frameshift	Class 5				51.5	3
Colorectal cancer	11	58892376	*FAM111B*	c.816dup	p.Ala273Serfs*9	Frameshift	Class 5				Variant not called	3
Colorectal cancer	11	58892522	*FAM111B*	c.952G>T	p.Glu318Ter	Nonsense	Class 5				86.2	3
Colorectal cancer	15	31214513	*FAN1*	c.2128C>T	p.Arg710Ter	Nonsense	Class 5		0.000069	0.000090	32.3	2
Colorectal cancer	15	31197237	*FAN1*	c.372_375dup	p.Ser126Aspfs*6	Frameshift	Class 5		0.000004	0.000009	53.9	2
Colorectal cancer	16	89865605	*FANCA*	c.862G>T	p.Glu288Ter	Nonsense	Class 5		0.000061	0.000117	21.4	2
Colorectal cancer	3	10133885	*FANCD2*	c.3799del	p.Tyr1267Thrfs*15	Frameshift	Class 5		0.000012	0.000027	45.0	3
Colorectal cancer	14	45628358	*FANCM*	c.1456C>T	p.Arg486Ter	Nonsense	Class 5				90.9	3
Colorectal cancer	14	45658326	*FANCM*	c.5101C>T	p.Gln1701Ter	Nonsense	Class 5		0.001289	0.000996	55.5	3
Colorectal cancer	9	20820943	*FOCAD*	c.1666C>T	p.Arg556Ter	Nonsense	Class 5		0.000049	0.000045	47.4	2
Colorectal cancer	17	29683996	*NF1*	c.7757C>A	p.Ser2586Ter	Nonsense	Class 5				6.6	3
Colorectal cancer	13	37439866	*SMAD9*	c.811C>T	p.Gln271Ter	Nonsense	Class 5				32.5	3
Colorectal cancer	8	30938648	*WRN*	c.1105C>T	p.Arg369Ter	Nonsense	Class 5		0.000179	0.000243	56.7	2
Colorectal cancer	3	37090053	*MLH1*	c.1942C>T	p.Pro648Ser	Missense	Class 5	C65			73.7	1
CUP	13	32912337	*BRCA2*	c.3847_3848del	p.Val1283Lysfs*2	Frameshift	Class 5		0.000045	0.000095	26.7	3
CUP	13	32914569	*BRCA2*	c.6082_6086del	p.Glu2028Lysfs*19	Frameshift	Class 5		0.000004		54.8	3
CUP	22	29091856	*CHEK2*	c.1100del	p.Thr367Metfs*15	Frameshift	Class 5		0.002077	0.002548	55.8	3
CUP	22	29121230	*CHEK2*	c.444+1G>A		Splice site	Class 5		0.000134	0.000188	44.4	3
CUP	5	138266225	*CTNNA1*	c.2074C>T	p.Gln692Ter	Nonsense	Class 5				51.5	3
CUP	9	98011506	*FANCC*	c.67del	p.Asp23Ilefs*23	Frameshift	Class 5		0.000130	0.000287	73.6	3
Endometrial cancer	17	41243837	*BRCA1*	c.3710del	p.Ile1237Asnfs*27	Frameshift	Class 5				60.2	2
Gastric cancer	9	101570214	*GALNT12*	c.239_243dup	p.Val82Argfs*63	Frameshift	Class 5				47.6	3
Head and Neck cancer	4	84391468	*ABRAXAS1*	c.364C>T	p.Gln122Ter	Nonsense	Class 5		0.000033	0.000072	65.7	3
Head and Neck cancer	16	89815091	*FANCA*	c.3323del	p.Phe1108Serfs*9	Frameshift	Class 5				43.8	2
Hepatocellular carcinoma	22	29091856	*CHEK2*	c.1100del	p.Thr367Metfs*15	Frameshift	Class 5		0.002077	0.002548	55.6	3
Hepatocellular carcinoma	3	14200140	*XPC*	c.1243C>T	p.Arg415Ter	Nonsense	Class 5		0.000012		44.6	3
Melanoma	11	108236087	*ATM*	c.9023G>A	p.Arg3008His	Missense	Class 4	C0	0.000008	0.000009	40.0	2
Melanoma (uveal)	3	52437907	*BAP1*	c.1254T>A	p.Tyr418Ter	Nonsense	Class 5				87.1	1
Mesothelioma	3	52437529	*BAP1*	c.1393_1421del	p.Ile465Glyfs*17	Frameshift	Class 5				Variant not called	1
Mesothelioma	13	32936732	*BRCA2*	c.7878G>C	p.Trp2626Cys	Missense	Class 5	C65	0.000004	0.000009	42.9	3
Mesothelioma	16	89828402	*FANCA*	c.2807A>G	p.Glu936Gly	Missense	Class 4	C0	0.000061	0.000134	41.4	2
Mesothelioma	9	98011506	*FANCC*	c.67del	p.Asp23Ilefs*23	Frameshift	Class 5		0.000130	0.000287	36.8	2
Mesothelioma	3	10133885	*FANCD2*	c.3799del	p.Tyr1267Thrfs*15	Frameshift	Class 5		0.000012	0.000027	44.1	2
Mesothelioma	14	45667921	*FANCM*	c.5791C>T	p.Arg1931Ter	Nonsense	Class 5		0.001028	0.001102	17.8	2
Mesothelioma	3	14200140	*XPC*	c.1243C>T	p.Arg415Ter	Nonsense	Class 5		0.000012		45.7	3
Neuroendocrine cancer	2	128047035	*ERCC3*	c.700C>T	p.Arg234Ter	Nonsense	Class 5				59.7	3
NSCLC	15	91328183	*BLM*	c.2695C>T	p.Arg899Ter	Nonsense	Class 5		0.000061	0.000134	57.3	3
NSCLC	22	29091856	*CHEK2*	c.1100del	p.Thr367Metfs*15	Frameshift	Class 5		0.002077	0.002548	57.1	3
NSCLC	22	29121230	*CHEK2*	c.444+1G>A		Splice site	Class 5		0.000134	0.000188	45.8	3
NSCLC	8	90990521	*NBN*	c.511A>G	p.Ile171Val	Missense	Class 4	C0	0.001211	0.001980	67.4	3
Oesophageal cancer	11	108143447	*ATM*	c.3154-2A>G		Splice site	Class 5				50.0	3
Oesophageal cancer	13	32913558	*BRCA2*	c.5073dup	p.Trp1692Metfs*3	Frameshift	Class 5		0.000018	0.000028	63.9	3
Others	10	59956091	*IPMK*	c.993_996del	p.Ser331Argfs*4	Frameshift	Class 5				Variant not called	3
Ovarian cancer	11	108192069	*ATM*	c.6498_6499del	p.Tyr2167Phefs*7	Frameshift	Class 5				45.4	3
Ovarian cancer	3	142274739	*ATR*	c.2320dup	p.Ile774Asnfs*3	Frameshift	Class 5				Variant not called	3
Ovarian cancer	17	41234476	*BRCA1*	c.4301del	p.Ser1434Metfs*22	Frameshift	Class 5				74.1	1
Ovarian cancer	13	32929057	*BRCA2*	c.7069_7070del	p.Leu2357Valfs*2	Frameshift	Class 5		0.000020	0.000045	62.5	1
Ovarian cancer	13	32953653	*BRCA2*	c.8953+1G>T		Splice site	Class 5				10.8	1
Ovarian cancer	16	89815091	*FANCA*	c.3323del	p.Phe1108Serfs*9	Frameshift	Class 5				48.1	3
Ovarian cancer	11	94180454	*MRE11*	c.1714C>T	p.Arg572Ter	Nonsense	Class 5		0.000061	0.000126	43.8	3
Pancreatic cancer	2	30142996	*ALK*	c.530G>A	p.Trp177Ter	Nonsense	Class 5				41.8	3
Pancreatic cancer	15	91328183	*BLM*	c.2695C>T	p.Arg899Ter	Nonsense	Class 5		0.000061	0.000134	28.0	2
Pancreatic cancer	17	41245991	*BRCA1*	c.1556del	p.Lys519Argfs*13	Frameshift	Class 5				59.6	1
Pancreatic cancer	13	32930747	*BRCA2*	c.7617+1G>A		Splice site	Class 5				58.0	1
Pancreatic cancer	14	45658326	*FANCM*	c.5101C>T	p.Gln1701Ter	Nonsense	Class 5		0.001289	0.000996	55.0	2
Pancreatic cancer	16	23640524	*PALB2*	c.2586+1G>A		Splice site	Class 5				51.6	1
Pancreatic cancer	5	131951698	*RAD50*	c.3040C>T	p.Gln1014Ter	Nonsense	Class 5				32.7	3
Pancreatic cancer	17	7578175	*TP53*	c.672+2T>G		Splice site	Class 5				63.2	1
Pancreatic cancer	8	30938648	*WRN*	c.1105C>T	p.Arg369Ter	Nonsense	Class 5		0.000179	0.000243	85.1	3
Prostate cancer	13	32914859	*BRCA2*	c.6373del	p.Thr2125Profs*12	Frameshift	Class 5				84.0	1
Prostate cancer	15	31200455	*FAN1*	c.1369C>T	p.Gln457Ter	Nonsense	Class 5		0.000030		53.9	3
Prostate cancer	8	90994963	*NBN*	c.156_157del	p.Ser53Cysfs*9	Frameshift	Class 5		0.000020	0.000036	41.7	3
Renal cell carcinoma	22	29091856	*CHEK2*	c.1100del	p.Thr367Metfs*15	Frameshift	Class 5		0.002077	0.002548	54.5	1
Renal cell carcinoma	20	18523722	*SEC23B*	c.1571C>T	p.Ala524Val	Missense	Class 4	C0	0.000020	0.000009	51.4	3

Sarcoma	11	125495891	*CHEK1*	c.236G>A	p.Trp79Ter	Nonsense	Class 5		0.001365	0.002290	66.7	3
Sarcoma	8	31012142	*WRN*	c.3690_3693del	p.Asp1231Serfs*16	Frameshift	Class 5		0.000004	0.000009	14.8	2
Sarcoma	3	14209830	*XPC*	c.463C>T	p.Arg155Ter	Nonsense	Class 5		0.000016	0.000027	49.4	3
Thymoma	15	91346889	*BLM*	c.3499del	p.Ala1167Argfs*5	Frameshift	Class 5				35.0	3
Urothelial cancer	15	91328183	*BLM*	c.2695C>T	p.Arg899Ter	Nonsense	Class 5		0.000061	0.000134	No tumor sample	3
Urothelial cancer	13	32900419	*BRCA2*	c.516G>A	p.Lys172Lys	Splice site	Class 5				60.6	3
Urothelial cancer	11	47254482	*DDB2*	c.574C>T	p.Arg192Ter	Nonsense	Class 5		0.000061		27.0	3
Urothelial cancer	9	101589120	*GALNT12*	c.628C>T	p.Arg210Ter	Nonsense	Class 5				45.1	3
Urothelial cancer	3	37081786	*MLH1*	c.1667+2_1667+8delinsATTT		Splice site	Class 5				No tumor sample	1
Urothelial cancer	2	47657006	*MSH2*	c.1203dup	p.Gln402Thrfs*15	Frameshift	Class 5				46.6	1
Vulvovaginal	7	6026390	*PMS2*	c.2006G>T	p.Ser669Ile	Missense	Class 4	C15			Variant not called	3

CUP, Cancer of Unknown Primary
GnomAD, The Genome Aggregation Database
NFE, non-Finish Europeans
AF, allele frequency

Supplementary Table 5. Patients (n=36) were selected for further evaluation.

Cancer type	Chr	Position	Gene	Nucleotide change	Protein change	Prior cancer	Known in patient	ACMG/AMP	Return	Treatment target
Mesothelioma	3	52437739	*BAP1*	c.1393_1421del	p.Ile465Glyfs*17	No	No	No	No[2]	No
Pancreatic cancer	17	7578175	*TP53*	c.672+2T>G		Yes	No[1]	Yes	Yes	No
Colorectal cancer	17	29683996	*NF1*	c.7757C>A	p.Ser2586Ter	No	No	No	Yes	No
CUP	13	32912337	*BRCA2*	c.3847_3848del	p.Val1283Lysfs*2	No	No	Yes	Yes	Yes
Breast cancer	17	41245072	*BRCA1*	c.2475del	p.Asp825Glufs*21	No	Yes	Yes	Yes	Yes
Ovarian cancer	13	32953653	*BRCA2*	c.8953+1G>T		No	Yes	Yes	Yes	Yes
Mesothelioma	13	32936732	*BRCA2*	c.7878G>C	p.Trp2626Cys	No	No	Yes	Yes	Yes
Pancreatic cancer	16	23640524	*PALB2*	c.2586+1G>A		No	No	No	No	No
Colorectal cancer	13	32911322	*BRCA2*	c.2830A>T	p.Lys944Ter	Yes	No	Yes	Yes	Yes
Pancreatic cancer	13	32930747	*BRCA2*	c.7617+1G>A		No	No	Yes	Yes	Yes
Pancreatic cancer	17	41245991	*BRCA1*	c.1556del	p.Lys519Argfs*13	Yes	Yes	Yes	Yes	Yes
Urothelial cancer	2	47657006	*MSH2*	c.1203dup	p.Gln402Thrfs*15	Yes	Yes	Yes	Yes	Yes
Prostate cancer	13	32914859	*BRCA2*	c.6373del	p.Thr2125Profs*12	No	No	Yes	Yes	Yes
Adenoid cystic carcinoma	17	41215947	*BRCA1*	c.5096G>A	p.Arg1699Gln	No	No	No	No[3]	No
Ovarian cancer	17	41234476	*BRCA1*	c.4301del	p.Ser1434Metfs*22	No	Yes	Yes	Yes	Yes
Colorectal cancer	3	37090053	*MLH1*	c.1942C>T	p.Pro648Ser	No	Yes	Yes	Yes	Yes
Ovarian cancer	13	32929057	*BRCA2*	c.7069_7070del	p.Leu2357Valfs*2	No	Yes	Yes	Yes	Yes
Breast cancer	16	68772227	*CDH1*	c.76G>T	p.Glu26Ter	Yes	No	No	No[2]	No
Bile duct cancer	17	59853761	*BRIP1*	c.2097+1G>C		No	No	No	No	No
Endometrial cancer	17	41243837	*BRCA1*	c.3710del	p.Ile1237Asnfs*27	No	Yes	Yes	Yes	Yes
Breast cancer	13	32911278	*BRCA2*	c.2787dup	p.Tyr930Ilefs*6	No	Yes	Yes	Yes	Yes
Breast cancer	13	32911297	*BRCA2*	c.2808_2811del	p.Ala938Profs*21	No	Yes	Yes	Yes	Yes
Breast cancer	13	32953947	*BRCA2*	c.9016_9017del	p.Tyr3006Glnfs*11	Yes	Yes	Yes	Yes	Yes
Urothelial cancer	13	32900419	*BRCA2*	c.516G>A	p.Lys172Lys	Yes	No	Yes	Yes	Yes
Breast cancer	17	41245721	*BRCA1*	c.1823_1826del	p.Lys608Ilefs*3	No	Yes	Yes	Yes	Yes
Oesophageal cancer	13	32913558	*BRCA2*	c.5073dup	p.Trp1692Metfs*3	Yes	Yes	Yes	Yes	Yes
Melanoma	3	52437907	*BAP1*	c.1254T>A	p.Tyr418Ter	No	No	No	No[2]	No
Bile duct cancer	17	41226347	*BRCA1*	c.4675+1G>A		No	No	Yes	Yes	Yes
Breast cancer	13	32914859	*BRCA2*	c.6373del	p.Thr2125Profs*12	No	Yes	Yes	Yes	Yes
Bile duct cancer	13	32928997	*BRCA2*	c.7008-1G>A		No	No	Yes	Yes	Yes
Colorectal cancer	17	59861629	*BRIP1*	c.1628+2T>G		No	No	No	No	No
CUP	13	32914569	*BRCA2*	c.6082_6086del	p.Glu2028Lysfs*19	Yes	No	Yes	Yes	Yes
Urothelial cancer	3	37081786	*MLH1*	c.1667+2_1667+8delinsATTT		No	No	Yes	Yes	Yes
Vulvovaginal	7	6026390	*PMS2*	c.2006G>T	p.Ser669Ile	No	No	Yes	No[4]	Yes
Bile duct cancer	16	23652477	*PALB2*	c.2T>C	p.Met1?	No	No	No	No	No
Colorectal cancer	16	68855903	*CDH1*	c.1712-1G>T		No	No	No	No	No

[1] Subjected to further germline analysis during the CoPPO project. The pathogenic germline variant was found and returned before this retrospective analysis

[2] Return of results to patient if alive according to consent

[3] No return was recommended in this case since the *BRCA1* variant p.Arg1699Gln is a moderate risk variant

[4] No return was recommended in this case due to recent evidence

CUP, Cancer of Unknown Primary

HGVS, Human Genome Variation Society

ACMG/AMP, The American College of Medical Genetics and Genomics / Association for Molecular Pathology
