# Supplementary material for: High frequency of pathogenic germline variants within homologous recombination repair in patients with advanced cancer
Source: NPJ Genom Med. 2019 Jun 21;4:13. doi: 10.1038/s41525-019-0087-6 (PMC6588611; doi:10.1038/s41525-019-0087-6)
Supplement: Supplementary file 2 — Reporting Summary Checklist [file 41525_2019_87_MOESM2_ESM.pdf]

nature research

Corresponding author(s): Finn Cilius Nielsen

Last updated by author(s): Apr 30, 2019

Reporting Summary

Nature Research wishes to improve the reproducibility of the work that we publish. This form provides structure for consistency and transparency in reporting. For further information on Nature Research policies, see Authors & Referees and the Editorial Policy Checklist.

Statistics

For all statistical analyses, confirm that the following items are present in the figure legend, table legend, main text, or Methods section.

n/a	Confirmed	
☒	☐	The exact sample size (*n*) for each experimental group/condition, given as a discrete number and unit of measurement
☒	☐	A statement on whether measurements were taken from distinct samples or whether the same sample was measured repeatedly
☒	☐	The statistical test(s) used AND whether they are one- or two-sided<br>*Only common tests should be described solely by name; describe more complex techniques in the Methods section.*
☒	☐	A description of all covariates tested
☒	☐	A description of any assumptions or corrections, such as tests of normality and adjustment for multiple comparisons
☒	☐	A full description of the statistical parameters including central tendency (e.g. means) or other basic estimates (e.g. regression coefficient) AND variation (e.g. standard deviation) or associated estimates of uncertainty (e.g. confidence intervals)
☒	☐	For null hypothesis testing, the test statistic (e.g. *F*, *t*, *r*) with confidence intervals, effect sizes, degrees of freedom and *P* value noted<br>*Give P values as exact values whenever suitable.*
☒	☐	For Bayesian analysis, information on the choice of priors and Markov chain Monte Carlo settings
☒	☐	For hierarchical and complex designs, identification of the appropriate level for tests and full reporting of outcomes
☒	☐	Estimates of effect sizes (e.g. Cohen's *d*, Pearson's *r*), indicating how they were calculated

Our web collection on statistics for biologists contains articles on many of the points above.

Software and code

Policy information about availability of computer code

Data collection	N/A
Data analysis	Sequenced reads were trimmed and mapped to hg19/GRCh37 reference genome using BWA-MEM v0.7.12 software. Alignment quality control was performed with mosdepth v0.2.0. Alignment files were pre-processed with GATK v3.8.0 suite. For germline variant calling, alignment files were analyzed with GATK v3.8.0 suite's HaplotypeCaller. Somatic variant calling in tumor samples was performed by MuTect2 from GATK v3.8.0 suite. Called germline variants were filtered using Ingenuity Variant Analysis version 5.5. Expression signatures were analized using Qlucore Omics Explorer v.3.3.

For manuscripts utilizing custom algorithms or software that are central to the research but not yet described in published literature, software must be made available to editors/reviewers. We strongly encourage code deposition in a community repository (e.g. GitHub). See the Nature Research guidelines for submitting code & software for further information.

Data

Policy information about availability of data

All manuscripts must include a data availability statement. This statement should provide the following information, where applicable:

- Accession codes, unique identifiers, or web links for publicly available datasets
- A list of figures that have associated raw data
- A description of any restrictions on data availability

The authors confirm that all relevant data are included in the paper and its supplementary information files. Raw data are available upon request. Microarray data are uploaded to Gene Expression Omnibus (GEO) with accession number GSE131027.

Field-specific reporting

Please select the one below that is the best fit for your research. If you are not sure, read the appropriate sections before making your selection.

☒ Life sciences ☐ Behavioural & social sciences ☐ Ecological, evolutionary & environmental sciences

For a reference copy of the document with all sections, see nature.com/documents/nr-reporting-summary-flat.pdf

Life sciences study design

All studies must disclose on these points even when the disclosure is negative.

Sample size	Patients were consecutively included in the period from May 2013 – February 2018. Sample size is 636 patients. No case-control analysis were performed, therefore no sample size calculations were performed.
Data exclusions	No data were excluded.
Replication	All variants evaluated relevant to return to the patients or their families were verified with a different method (panel-based NGS).
Randomization	N/A
Blinding	N/A

Reporting for specific materials, systems and methods

We require information from authors about some types of materials, experimental systems and methods used in many studies. Here, indicate whether each material, system or method listed is relevant to your study. If you are not sure if a list item applies to your research, read the appropriate section before selecting a response.

Materials & experimental systems

n/a	Involved in the study	
☒	☐	Antibodies
☒	☐	Eukaryotic cell lines
☒	☐	Palaeontology
☒	☐	Animals and other organisms
☐	☒	Human research participants
☒	☐	Clinical data

Methods

n/a	Involved in the study	
☒	☐	ChIP-seq
☒	☐	Flow cytometry
☒	☐	MRI-based neuroimaging

Human research participants

Policy information about studies involving human research participants

Population characteristics	Patients with advanced solid cancers and exhausted treatment options were included in a prospective, single-center, single-arm open-label study (CoPPO study). Detailed characteristics of the patients in this study are presented in Table 1. E.g. number of patients = 636; 50% female; 50% male; median age 57 (range 16-82).
Recruitment	This study included a cohort of patients enrolled in the Copenhagen Prospective Personalized Oncology (CoPPO) study (NCT02290522) from May 2013 – February 2018.
Ethics oversight	Regulatory approvals from the Regional Ethics Committee and the Danish Data Protection Agency were obtained (Danish Ethical Committee, file number: 1300530)

Note that full information on the approval of the study protocol must also be provided in the manuscript.
